# *Blastocystis* infection in Tibetan antelopes (*Pantholops hodgsonii*) alters gut microbiota composition and function

**DOI:** 10.3389/fcimb.2025.1719025

**Published:** 2025-12-02

**Authors:** Jian Liu, Si-Yuan Qin, Cong-Cong Lei, He Ma, Lin-Hong Xie, Yan Liu, Jing-Hao Li, Hong-Bo Ni, Ming-Yuan Yu, Hong-Rui Liang, Wen-Hui Shi, Ya Qin, Jing Jiang, Wei-Lan Yan, Bei-Ni Chen, Zhong-Yuan Li, He-Ting Sun

**Affiliations:** 1 Guangxi Key Laboratory of Brain and Cognitive Neuroscience, College of Basic Medicine, Guilin Medical University, Guilin, Guangxi, China; 2 College of Life Sciences, Changchun Sci-Tech University, Jilin, Changchun, China; 3 College of Veterinary Medicine, Qingdao Agricultural University, Qingdao, Shandong, China; 4 Center of Prevention and Control Biological Disaster, State Forestry and Grassland Administration, Shenyang, Liaoning, China

**Keywords:** Tibetan antelope, gut microbiota, metagenome, function analysis, *Blastocystis* infection

## Abstract

**Introduction:**

The gut microbiota plays an important role in host environmental adaptation, including defense against pathogens. Parasite infections can disrupt gut microbial communities and thus influence host adaptability. However, most current knowledge of Blastocystis–microbiota interactions comes from humans or domestic animals, and data from wild mammals, especially those inhabiting extreme environments, remain scarce.

**Methods:**

In this study, we analyzed 68 gut metagenomes from Tibetan antelopes (Pantholops hodgsonii) and screened for infections by four intestinal parasites — Blastocystis, Cryptosporidium, Giardia, and Encephalitozoon bieneusi.

**Results:**

Among them, 26 individuals were solely infected with Blastocystis subtype ST31. Compositional analysis revealed 25 differential families, with 12 enriched in infected and 13 in healthy individuals. LEfSe further identified 38 species-level biomarkers (LDA > 2, p < 0.05), indicating a significant shift in gut microbial diversity following Blastocystis ST31 infection. Notably, the relative abundance of Arthrobacter sp. 08Y14, associated with environmental resilience, was markedly reduced in infected individuals. Functional profiling showed a decrease in metabolic diversity, with 18 CAZy families detected in the healthy group but only 2 in the infected group. KEGG analysis showed that the average relative abundance of K07497 was higher in the infected group (5.16) than in the healthy group (1.03).

**Discussion:**

These findings suggest that Blastocystis ST31 infection reshapes the gut microbiota and may impair the high-altitude adaptability of Tibetan antelopes by reducing plateau-adaptive microbes and functional capacity. This study provides the first evidence of Blastocystis-induced gut microbiota changes in Tibetan antelopes and broadens our understanding of parasite–microbiota interactions across hosts.

## Introduction

The gut microbiota plays a pivotal role in host health, forming a complex and dynamic ecosystem that influences numerous physiological processes, including nutrient absorption, energy metabolism, immune regulation, and environmental adaptation (Rooks and Garrett, 2016; De Vos et al., 2022). Functionally, the gut microbiome can be regarded as a “hidden organ” that co-evolves with its host, maintaining a delicate balance between stability and plasticity in response to environmental or dietary shifts (Kuziel and Rakoff-Nahoum, 2022). Beyond supporting digestion and nutrient assimilation, gut microorganisms produce a wide range of bioactive metabolites, such as short-chain fatty acids, bile acid derivatives, and neurotransmitter precursors, which can modulate host metabolic pathways, immune responses, and even behavior (Shi et al., 2017; Wang et al., 2021). A healthy and diverse microbial community is generally associated with host resilience, whereas dysbiosis has been linked to a variety of metabolic, inflammatory, and infectious diseases across mammals (De Vos et al., 2022). These microbial communities are especially critical under resource-limited or extreme ecological conditions, where they contribute to host survival by facilitating the degradation of indigestible plant polysaccharides, synthesizing essential vitamins and short-chain fatty acids, and modulating immune and antioxidant responses (Li and Zhao, 2015; Morita et al., 2019).

The Tibetan antelope (*Pantholops hodgsonii*), as a representative species of plateau-dwelling animals, is primarily distributed across alpine grasslands and desert regions at altitudes above 4,000 meters (Schaller et al., 1991). These habitats are characterized by extremely harsh environmental conditions, including persistently low temperatures, hypoxia, intense ultraviolet radiation, and sparse vegetation (Xu et al., 2005). To survive in such an extreme ecological environment, Tibetan antelopes have developed unique morphological, physiological, and behavioral adaptations through long-term evolution (Ge et al., 2013). These include physiological, cellular, and molecular features such as increased skin pigmentation (Wang et al., 2000), a well-developed cardiopulmonary system with a dense capillary network (Mitra et al., 1968; Scott et al., 2009), and upregulated expression of vascular endothelial growth factor (VEGF) (Nei and Gojobori, 1986). Such adaptations not only ensure their survival and reproduction but also make the Tibetan antelope an ideal model for studying the mechanisms of environmental adaptation in high-altitude animals.

Beyond environmental pressures, parasitic infections can further influence the structure and function of host gut microbiota. *Blastocystis* is one of the few intestinal parasites, commonly found in immunocompromised individuals such as those with human immunodeficiency virus/acquired immunodeficiency syndrome (HIV/AIDS) or cancer, and people with close contact with animals are at a higher risk of infection (Wawrzyniak et al., 2013). While traditionally considered a potential pathogen associated with gastrointestinal disorders (Bálint et al., 2014), increasing evidence suggests that certain subtypes of *Blastocystis* may coexist with the host as commensals or even contribute to maintaining microbial diversity and intestinal homeostasis (Scanlan et al., 2014; Audebert et al., 2016; Kodio et al., 2019; Even et al., 2021). Recent studies have demonstrated that *Blastocystis* infection can modulate functional metabolic pathways (Puthia et al., 2006; Puthia et al., 2008), and affect host immune responses (Puthia et al., 2008), thereby shaping the overall gut ecosystem. These effects, however, appear to be highly context-dependent, varying with host species, infection subtype, and environmental conditions. Despite these advances, little is known about how *Blastocystis* infection impacts the gut microbiota of high-altitude animals such as the Tibetan antelope.

Given the unique ecological challenges faced by Tibetan antelopes and the potential impact of *Blastocystis* infection on gut microbial communities, it is essential to investigate how such infections may reshape host–microbiota interactions under high-altitude conditions. In this study, we analyzed 68 fecal metagenomic samples from Tibetan antelopes, including 26 infected with *Blastocystis* and 42 uninfected individuals. Our objective was to characterize the diversity, structure, and functional potential of their gut microbiota, and to determine how *Blastocystis* infection may influence microbial community dynamics under high-altitude conditions. These findings provide new insights into host–microbiota interactions and offer a theoretical foundation for the conservation of plateau-dwelling wildlife.

## Materials and methods

### Sample collection and parasitic infection detection

In this study, Tibetan antelopes were randomly observed in the wild. Fresh fecal samples were collected immediately after defecation into PE gloves, placed on ice, and transported to the laboratory. A total of 68 samples were collected and screened for parasitic infections, including *Encephalitozoon bieneusi*, *Giardia*, *Cryptosporidium*, and *Blastocystis*. Among these, 26 samples were positive for *Blastocystis* infection—the only parasite detected—resulting in a prevalence of 38.2%, while the remaining 42 samples were uninfected. The detection method followed that described in a previous study (Geng et al., 2021; Yan et al., 2024). All samples were stored at −80°C until further processing.

### Metagenome sequencing

In this study, we isolated total DNA from 68 fecal samples using the OMEGA Mag-Bind Soil DNA Kit (M5635-02; Omega Bio-Tek, Norcross, GA, USA) according to the manufacturer’s protocol. DNA yield and purity were first evaluated with a Qubit™ 4 Fluorometer and further checked on 1% agarose gels. Only samples that passed quality control were normalized to 10 nM for library construction. Metagenomic libraries were then sequenced on an Illumina HiSeq platform with paired-end 2 × 150 bp reads, producing approximately 10 Gb of raw sequence data per sample. All raw sequences can be found in the National Center for Biotechnology Information (NCBI) database under accession number PRJNA1257558.

### Data processing

Fastp (v0.23.0) was used to filter for high-quality reads, which were then processed with Bowtie2 (v2.5.0) to remove host genomic (PHO1) DNA contamination. Contigs were assembled using MEGAHIT (v1.2.9) (Li et al., 2016). ORFs were predicted from the assembled contigs using Prodigal (v2.6.3). The resulting CDSs were clustered and de-redundantized using MMseqs with parameters “–cluster-mode 2 –min-seq-id 0.9 –cov-mode 2 -c 0.9”.

### Taxonomy analysis

Gut bacteriome composition was characterized from fecal metagenomic sequences using MetaPhlAn4 (Blanco-Míguez et al., 2023), a clade-specific marker-based taxonomic profiling approach. Species-level relative abundances were normalized for each sample, and genus- and phylum-level abundances were obtained by aggregating the corresponding species abundances.

### LEfSe analysis

Using LEfSe (Linear Discriminant Analysis Effect Size) (Segata et al., 2011), a computational approach for high-dimensional biomarker discovery and interpretation—we systematically identified taxonomic features distinguishing *Blastocystis*-infected individuals from uninfected controls. In the statistical analysis, the α values for the Kruskal–Wallis test between groups and the Wilcoxon rank-sum test between subgroups were both set to 0.05. The logarithmic LDA score threshold for discriminative features was set to 2.0. Biomarkers with significant LDA scores were further validated to assess their potential roles in the gut environment.

### Function annotation

Functional annotation of the clustered CDSs was performed by aligning them to the KEGG using DIAMOND (v0.9.22) with parameters –min-score 60 –query-cover 50. To explore carbohydrate metabolism capabilities, protein-coding genes were also annotated against the CAZyme database (Cantarel et al., 2009) using DIAMOND with parameters –min-score 60 –query-cover 50. For each ORF, the highest bit-score alignment was selected as the representative functional and taxonomic annotation.

### Data analysis and visualization

Statistical analyses were conducted in R (v4.2.2). To investigate factors influencing the gut microbiota composition of Tibetan antelopes, permutational multivariate analysis of variance (PERMANOVA) was performed based on a Bray–Curtis distance matrix. The model included three factors: sampling date, sampling location, and parasitic infection status. Two approaches were applied: the marginal effects method (by=“margin”) to assess the independent contribution of each factor, and the sequential method (by=“terms”) to evaluate the contributions of main effects and their interactions. Alpha diversity metrics (Richness, Shannon index) were computed from taxonomic and functional profiles. Beta diversity was assessed using Principal Coordinate Analysis (PCoA) based on Bray-Curtis dissimilarity, with permutational multivariate ANOVA (PERMANOVA) testing group differences. Wilcoxon rank-sum tests identified significant differences in diversity indices, taxa abundance, and functional gene profiles between groups. Visualizations employed the ggplot2 package (v4.2.3) for all other graphical representations.

### Correlation analysis

Multiple testing was corrected using the Benjamini–Hochberg FDR method, and correlations with an absolute Spearman’s coefficient > 0.8 and an adjusted p-value (q-value)<0.05 were considered significant.

## Results

### Comprehensive information of the Tibetan antelope dataset

**Figure 1:**
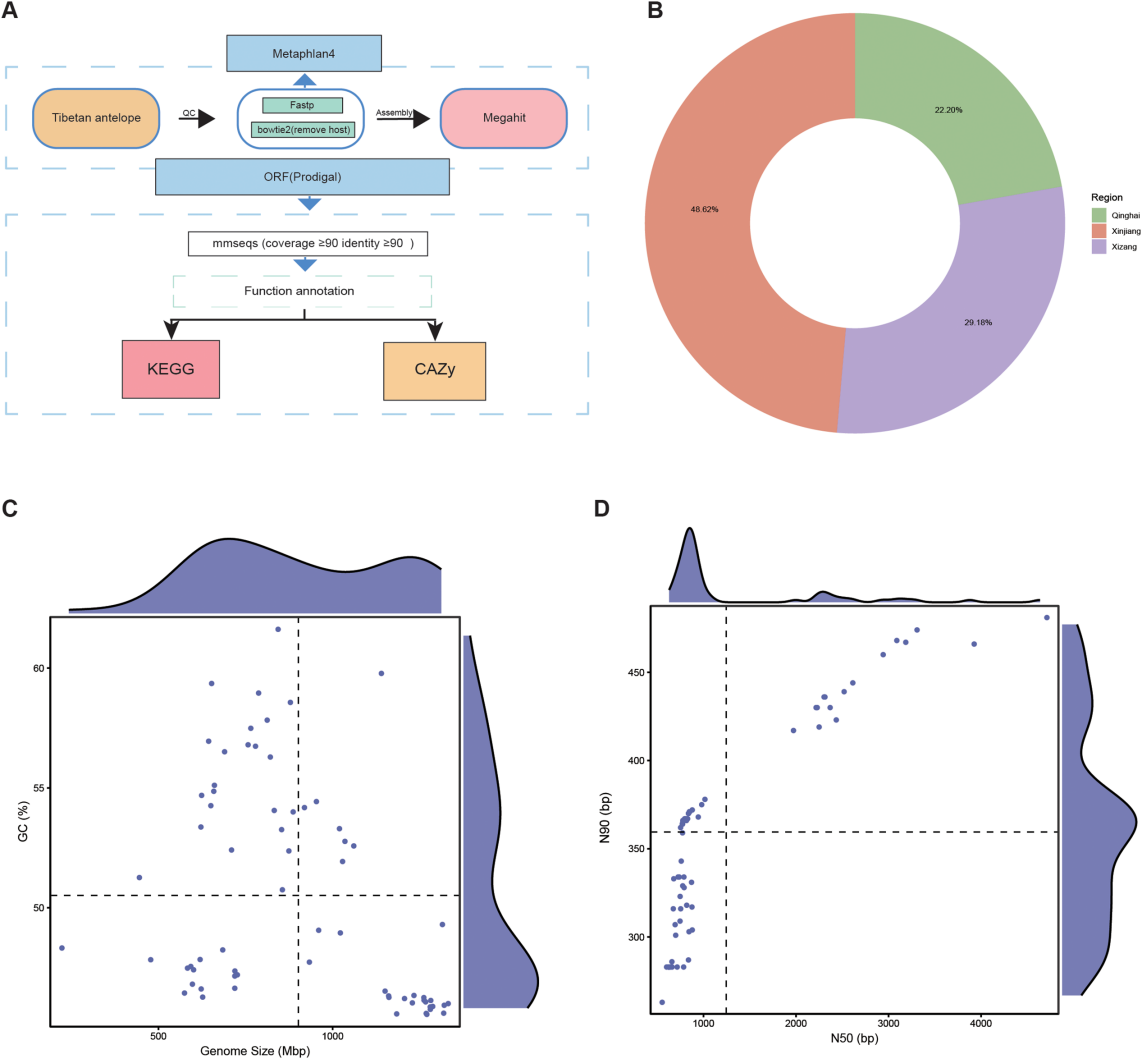
Metagenome information of Tibetan antelope **(A)** Standardized data processing pipeline. **(B)** Circular pie chart illustrating the regional distribution of the non-redundant gene set, with cyan for Qinghai, orange for Xinjiang, and purple for Xizang. **(C)** GC content and genome size distribution of the 68 Tibetan antelope samples. **(D)** N50 and N90 distribution.

After standardized processing of 68 Tibetan antelope gut metagenomes (**Figure 1A**), coding sequences predicted by Prodigal were clustered, yielding a total of 74,571,417 non-redundant genes (see Methods for details). Among them, the majority originated from the Xinjiang region (48.62%), while the proportions of non-redundant genes from the Qinghai (22.20%) and Xizang (29.18%) regions were comparable (**Figure 1B**).

Additionally, several characteristics of the assembled contigs were summarized. Contigs sizes ranged from 0.38 to 6.50 Mbp (average 1.89 Mbp/genome), while GC content varied between 23.93% and 73.57% (average 45.82%) (**Figure 1C**). The average N50 of these contigs was 82.50%, with a mean N90 of 1.95% (**Figure 1D**).

### Composition of Tibetan antelope gut microbiome

Taxonomic classification was performed using MetaPhlAn4. Analysis of the microbial composition revealed clear differences in taxa among different regions at the family level. Further analysis of Tibetan antelope gut microbiota from different regions revealed marked differences in community composition at the family level (**Supplementary Figure 1**). For example, Micrococcaceae and Bacteroidales_unclassified were dominant in both the Xinjiang and Xizang regions. However, substantial variation in gut microbial composition was also observed among individual samples within these regions. Two approaches were applied in the PERMANOVA analysis: a marginal-effects approach (by=“margin”) to evaluate the independent contribution of each factor (**Supplementary Figure 2A**), and a sequential approach (by=“terms”) to assess the contributions of main and interaction effects (**Supplementary Figure 2B**). Both approaches yielded essentially identical results. Both marginal and sequential analyses indicated that sampling location was the primary factor shaping the composition of gut microbiomes, with an adjusted R² of 0.45 (*p* =0.001), followed by sampling date (adjusted R²=0.21, *p*=0.001) and parasitic infection status (adjusted R²=0.07, *p*=0.001). These results suggest that the gut microbiota of Tibetan antelopes is strongly influenced by the characteristics of their specific habitats, while temporal variation also plays a significant role, potentially reflecting differences in regional antibiotic exposure and environmental conditions.

**Figure 2:**
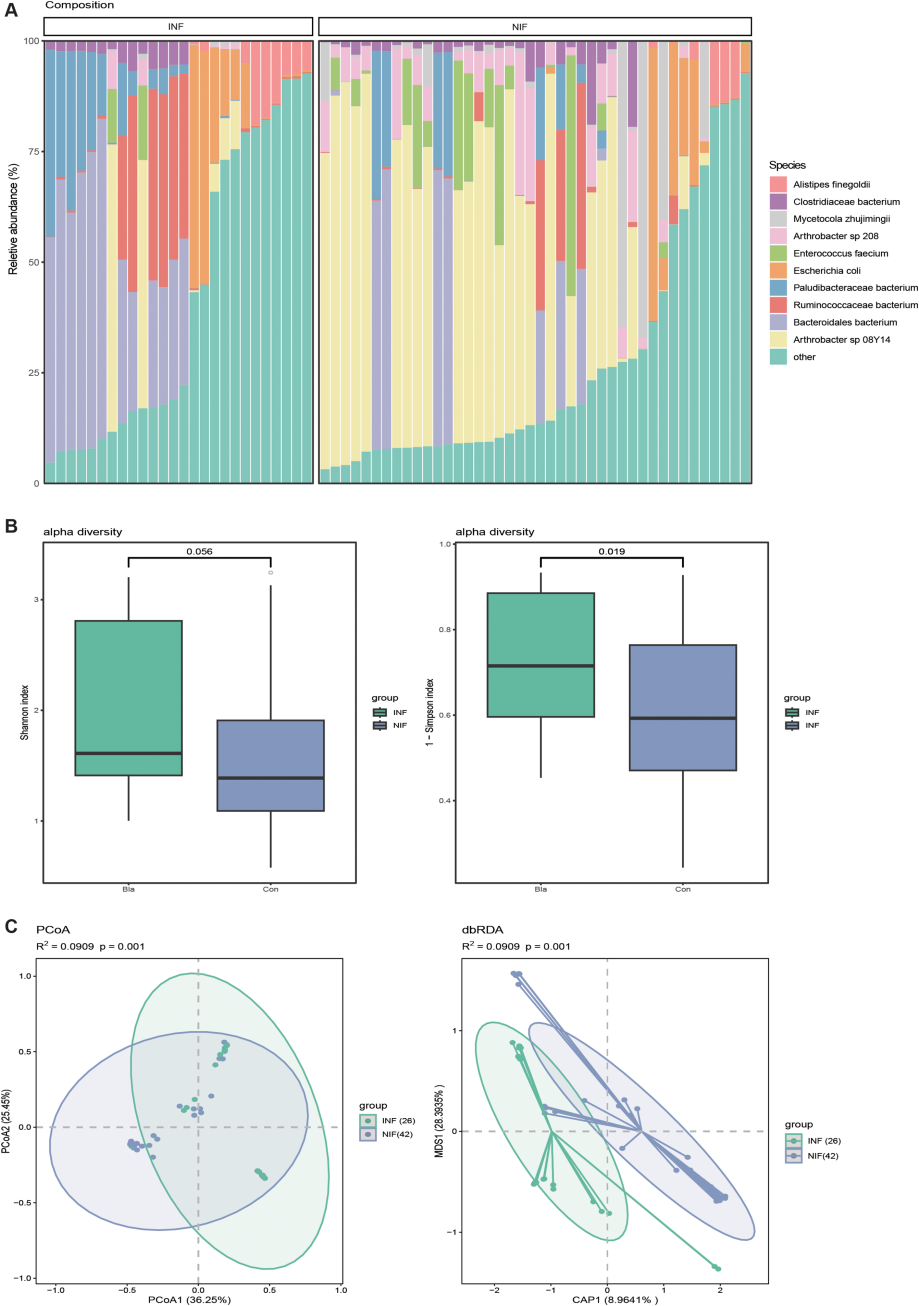
Gut microbial composition of Tibetan antelopes **(A)** Bacterial composition of the Tibetan antelope gut microbiota at the species level. The figure shows the top 10 most abundant species, with all remaining taxa grouped as “other.” NIF, uninfected group; INF, *Blastocystis*-infected group. **(B)** Boxplots illustrating species-level alpha diversity, with the left panel showing the Shannon index and the right panel showing the 1-Simpson index. **(C)** Principal Coordinates Analysis (PCoA) and Distance-based Redundancy Analysis (dbRDA) based on Bray-Curtis dissimilarity. PERMANOVA results show R² and p-values assessing group separation.

Among the 68 Tibetan antelope samples, 26 individuals were infected with *Blastocystis*, while 42 served as not infected. A comparative analysis of the gut microbiota was performed. Using the Wilcoxon rank-sum test, 25 bacterial families showing significant differences between the two groups (p<0.05) were identified, with 12 enriched in infected and 13 in healthy individuals(**Supplementary Figure 3**). At the species level, *Arthrobacter* sp. *08Y14* exhibited a significant difference in abundance, being markedly higher in the uninfected group compared to the infected group. Alpha diversity analysis revealed no significant difference in Shannon index between the two groups (p=0.056), while Simpson index showed a significant difference (p=0.019) (**Figure 2B**). Principal coordinate analysis (PCoA) indicated a clear separation between infected and non-infected samples (p=0.01, R²=0.09), suggesting distinct microbial community structures (**Figure 2C**).

### LEfSe analysis between infected and healthy Tibetan antelopes

**Figure 3:**
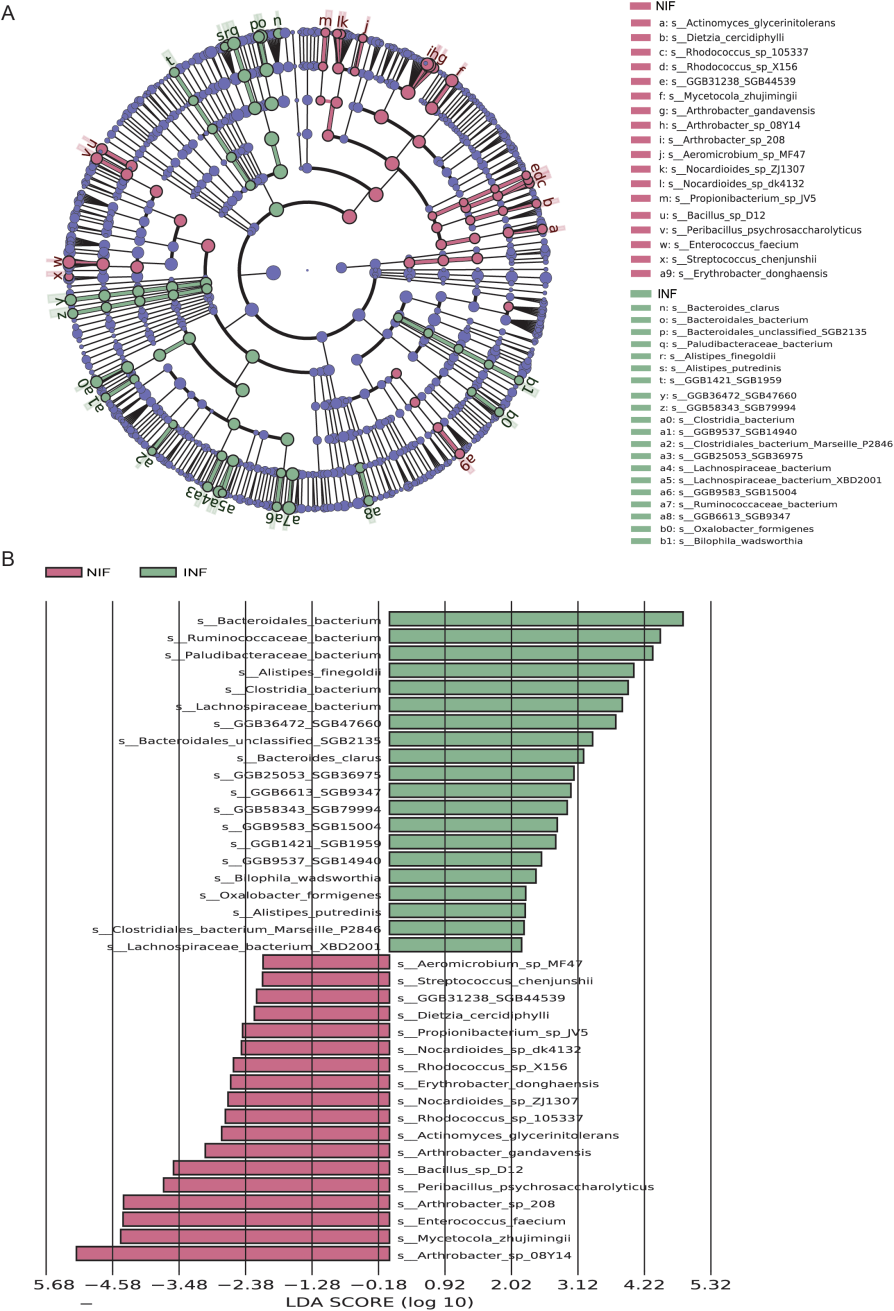
LEfSe analysis identifying marker taxa between the infected and uninfected groups. **(A)** Differential enrichment of gut bacteria taxa in infections and uninfected. The cladogram visualizes all differentially enriched taxa identified by LEfSe analysis. Each dot corresponds to a bacteria species taxon, with significant enrichments (p<0.05, LDA > 2.0) labeled in brown for infections and red for uninfected. **(B)** The bar plot at the bottom right displays the differentially enriched species (p<0.05, LDA > 2.0).

Further analysis using LEfSe identified 38 differential species between the infected and uninfected groups (**Figure 3A**). Among them, 13 belonged to the Firmicutes (accounting for 36.8%), followed by the Actinobacteria (34.2%). Consistent with the previous results, *Arthrobacter* sp. *08Y14* was the most significantly enriched taxon in the uninfected group (**Figure 3B**). In contrast, the most significantly enriched taxon in the infected group was *Bacteroidales bacterium*.

Subsequently, we classified these 38 taxa at the family and phylum levels to examine their distribution patterns (**Supplementary Figure 4**). Most of the taxa enriched in the uninfected group belonged to Actinobacteria, followed by Firmicutes (**Supplementary Figure 4B**). Among the taxa enriched in the infected group, a large proportion belonged to the genus Firmicutes, followed by Bacteroidetes (**Supplementary Figure 4D**). Consistently, the infected group exhibited an increased abundance of Firmicutes and Bacteroidetes, while Actinobacteria showed a decrease trend.

### Functional differences analysis in Tibetan antelopes

**Figure 4:**
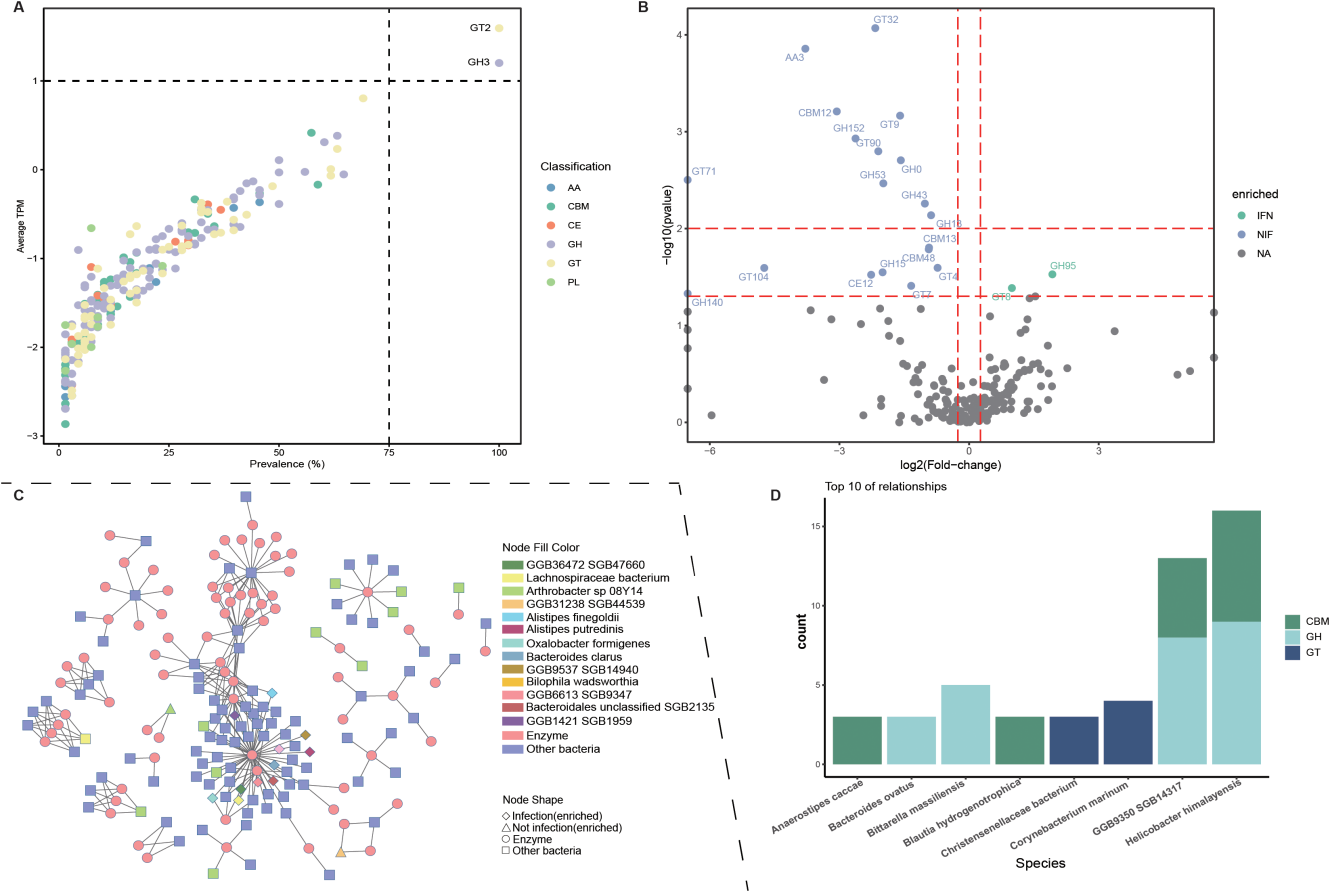
Functional analysis based on CAZy annotations **(A)** Prevalence and abundance distribution of all CAZy genes in the 68 samples. The abundance of each CAZy genes is represented as the mean value across all samples. Dots of different colors represent distribution patterns based on abundance levels and frequencies. **(B)** Volcano plot depicting fold changes and significance (*p*-values) of functional features in CAZy genes. Points represent features with absolute fold change > 1.5 and p-value<0.05; green dots indicate features enriched in infected group, purple dots indicate features enriched in uninfected group. **(C)** Correlation network showing species-level bacteria and CAZy enzymes with Spearman’s correlation coefficients (|r| > 0.8). Circles represent CAZy enzymes, squares represent bacterial species, and triangles and diamonds indicate the groups in which these bacteria are enriched. **(D)** Stacked bar chart showing the top eight bacterial species with the highest number of correlations and their associated CAZy enzymes.

CAZy analysis revealed that GT2(Glycosyl Transferase 2) and GH3(Glycosyl Transferase 3) were the two most prevalent CAZy enzyme families (**Figure 4A**). In the infected group, the enriched enzymes primarily belonged to the GH (Glycoside Hydrolase) and GT (Glycosyl Transferase) families. In contrast, the uninfected group exhibited greater enzymatic diversity, with the majority of enriched enzymes associated with the GT and GH families, followed by the CBM (Carbohydrate-Binding Module) family (**Figure 4B**). Furthermore, correlation analysis at the species level identified bacterial taxa with strong associations, defined by an absolute correlation coefficient greater than 0.8 (**Figure 4C**).

In addition, KEGG analysis was performed to investigate the KO pathways enriched in the gut microbiota of Tibetan antelopes. Genes with TPM values less than 100 were removed during profile table construction. Consequently, 13 samples exhibited no detectable KEGG abundance and were excluded from further analysis. The most abundant KO in the Tibetan antelope gut was K00973, an enzyme involved in the biosynthesis of streptomycin, polyketide sugar units, and acarbose and validamycin, followed by K07486 and K07485 (**Supplementary Figure 5B**). Subsequent group comparison revealed that the K07497 pathway was more abundant in the infected group and downregulated in the uninfected group. K07497 encodes a putative transposase, which may be associated with the horizontal transfer of certain genes(**Supplementary Figure 5A**).

## Discussion

Due to the unique environmental conditions of the plateau, such as low temperature, hypoxia, and intense ultraviolet radiation, plateau animals have evolved a series of adaptations to counteract the harsh environment (Ma et al., 2019). For example, Tibetan antelopes have developed specialized adaptive mechanisms to mitigate these adverse effects. Although, in recent years, multi-dimensional studies have been conducted on the high-altitude adaptation mechanisms of native animals on the Qinghai–Tibet Plateau, research on the microbiota—particularly the gut microbiota—that co-evolve with the host and play pivotal roles in metabolic, digestive, and immune functions remains limited (Turnbaugh et al., 2006; Tremaroli and Bäckhed, 2012). Therefore, this study used the gut microbiota as an entry point to examine the composition and functional traits of the Tibetan antelope gut microbial community, aiming to elucidate its role in adaptation to extreme environments. Previous studies have analyzed the gut microbiota composition of Tibetan antelopes using a macro-taxonomic approach based on operational phylogenetic units and near full-length PacBio amplicon sequences. These studies found that both the dominant and core bacterial taxa in the fecal microbiome were largely composed of unknown species, with several taxa potentially representing novel genera within their respective families (Turnbaugh et al., 2006). This observation is consistent with our MetaPhlAn4-based mapping results, in which a substantial proportion of species could not be taxonomically assigned. And the 68 Tibetan antelope gut microbiomes exhibited pronounced regional variation, likely driven by environmental differences among sampling locations. Although both Xizang and Qinghai are situated on the plateau, their considerable geographic separation results in distinct climatic, vegetational, and other ecological conditions, which in turn shape divergent microbial community structures (Chen et al., 2022).

*Blastocystis* is a common protozoan widely distributed in the gastrointestinal tracts of humans and various animal species worldwide, exhibiting high genetic diversity (Rauff-Adedotun et al., 2021). It colonizes the gastrointestinal tract; however, its mechanisms of action in relation to gut health and disease remain poorly understood (Andersen and Stensvold, 2016). In our study, infection was associated with a marked shift in gut microbial composition, characterized by an increased relative abundance of Firmicutes and Bacteroidetes and a concomitant decrease in Actinobacteria. Such phylum-level alterations have been widely reported in infection- and inflammation-related dysbiosis, where changes in nutrient availability, gut pH, and immune status collectively reshape the microbial ecosystem (Trebicka et al., 2021; Mantovani et al., 2024). The enrichment of Bacteroidales bacterium in the infected group may reflect its metabolic versatility, particularly in carbohydrate degradation, which can confer a competitive advantage under altered gut conditions. Conversely, the uninfected group was enriched in *Arthrobacter* sp. *08Y14*. Previous studies have indicated that *Arthrobacter* spp. exhibit adaptive traits enabling their survival in polar and alpine environments (Shen et al., 2019). The observed reduction in Actinobacteria, may indicate a loss of protective functions, potentially compromising gut barrier integrity and anti-inflammatory capacity (Huang et al., 2019). Together, these findings suggest that infection induces a restructuring of the gut microbial community that could influence both host metabolism and immune responses.

Furthermore, GT2 and GH3 emerged as the most prevalent CAZy enzyme families in the Tibetan antelope gut microbiota. The GT2 family is large, encompassing enzymes encoded by the cellulose synthase superfamily as well as numerous other transferases with diverse substrate specificities, and is widely distributed from archaea to humans (Stone et al., 2010). This prevalence may imply that Tibetan antelopes rely on these enzymes to efficiently synthesize and modify polysaccharides, facilitating the digestion of complex plant materials and supporting energy acquisition in their nutrient-limited, high-altitude habitats. And KEGG functional profiling further revealed that K00973, associated with the biosynthesis of streptomycin, polyketide sugar units, and acarbose/validamycin (Jiang et al., 1991), was the most abundant KO, suggesting an active microbial capacity for secondary metabolite production.

Overall, in addition to deepening our understanding of gut microbiota–parasite interactions in Tibetan antelope, our findings also have practical implications for conservation management. For example, changes in *Blastocystis* infection rates or accompanying alterations in gut microbial diversity may indicate shifts in environmental conditions or increased frequency of contact with livestock and humans (Yoshikawa et al., 2004; Ithoi et al., 2011). Moreover, when Blastocystis subtypes carried by Tibetan antelope overlap with those reported in domestic animals, these distribution patterns can help managers identify high-risk interface areas and prioritize the establishment of buffer zones to separate wildlife and livestock activity ranges. Therefore, long-term monitoring of *Blastocystis* along migration routes may contribute to the development of an early-warning system for emerging health threats and support adaptive management strategies aimed at maintaining the resilience of Tibetan antelope populations.

In addition, this study has several limitations. First, the overall sample size was relatively small, which may not fully capture the gut microbiota composition of the broader Tibetan antelope population. Moreover, the number of samples collected from each region was limited; although significant differences were observed, these findings may be influenced by sampling variability. Second, the proportion of Blastocystis-infected samples relative to uninfected controls was imbalanced, potentially affecting the robustness of comparative analyses. Third, a substantial fraction of the gut microbiota could not be taxonomically classified to precise levels, limiting detailed insights into community structure and functional potential. Although we provide valuable insights into the microbiota differences associated with infection status, the cross-sectional design does not allow us to directly track how infections influence the microbiota over time within the same individuals. Finally, the cross-sectional nature of the dataset precludes assessment of temporal dynamics, and environmental variables in the Tibetan antelopes’ natural habitats—such as ambient temperature, dietary composition, and seasonal variation—were not monitored, all of which may influence microbial composition and function. Future studies incorporating larger, balanced, longitudinal datasets, multi-kingdom microbial profiling, and environmental monitoring will be essential to fully elucidate the role of the gut microbiota in high-altitude adaptation.

## Conclusion

In summary, this study provides the first comprehensive evidence that *Blastocystis* infection drives taxon- and function-specific alterations of the Tibetan antelope gut microbiota, with potential consequences for host adaptation to high-altitude environments. The marked reduction of *Arthrobacter* sp. 08Y14 and a *Bacteroidales* bacterium indicates the loss of microbial taxa likely important for environmental resilience. Functionally, while K00973, the most abundant KO involved in antibiotic and carbohydrate biosynthesis, underscores the intrinsic role of gut microbes in mediating microbial competition and energy balance in Tibetan antelopes, *Blastocystis* infection was associated with reduced metabolic versatility, as reflected by a narrower repertoire of CAZy. This functional contraction suggests impaired polysaccharide degradation and energy harvest, potentially weakening the host’s capacity to cope with nutritional constraints under extreme plateau conditions. By linking protozoan infection to specific microbial losses and constrained metabolic potential, this study advances understanding of host–microbe–protist interactions and reveals a novel mechanism by which gut eukaryotes may influence wildlife adaptation in harsh environments.

## Data Availability

The datasets presented in this study can be found in online repositories. The names of the repository/repositories and accession number(s) can be found in the article/**Supplementary Material**.

## Supplementary material

Supplementary Figure 1 Family-level composition of the Tibetan antelope gut microbiome.

Supplementary Figure 2 Permutational multivariate analysis of variance (PERMANOVA) based on Bray – Curtis distance matrices. Two approaches were applied: (A) the marginal effects method (by=“margin”) to assess the independent contribution of each factor, and (B) the sequential method (by=“terms”) to evaluate the contributions of main effects and interactions. * *P*<0.05; ** *P*<0.01.

Supplementary Figure 3 Differential bacterial families (Wilcoxon *p*<0.05) between the infected and uninfected groups.

Supplementary Figure 4 Distribution proportions of differential bacteria at the family and phylum levels. **(A-B)** Uninfected group. (C-D) Infected group.

Supplementary Figure 5 KO composition. **(A)** Composition of relative abundances of KO pathways. **(B)** Overall composition proportions of KO pathways.

Supplementary Table 1 Blastocystis infection status of the sampled Tibetan antelopes.

Supplementary Table 2 Composition at species level in Tibetan antelope gut.

Supplementary Table 3 Composition at family level in Tibetan antelope gut.

Supplementary Table 4 Difference between uninfected group and infected group in Tibetan antelope.

Supplementary Table 5 Composition of CAZy enzyme in Tibetan antelope.

Supplementary Table 6 Composition of KOs in Tibetan antelope.
