# Supplementary figures and images for: Blastocystis infection in Tibetan antelopes (Pantholops hodgsonii) alters gut microbiota composition and function
Source: Front Cell Infect Microbiol. 2025 Dec 2;15:1719025. doi: 10.3389/fcimb.2025.1719025 (PMC12705584; doi:10.3389/fcimb.2025.1719025)

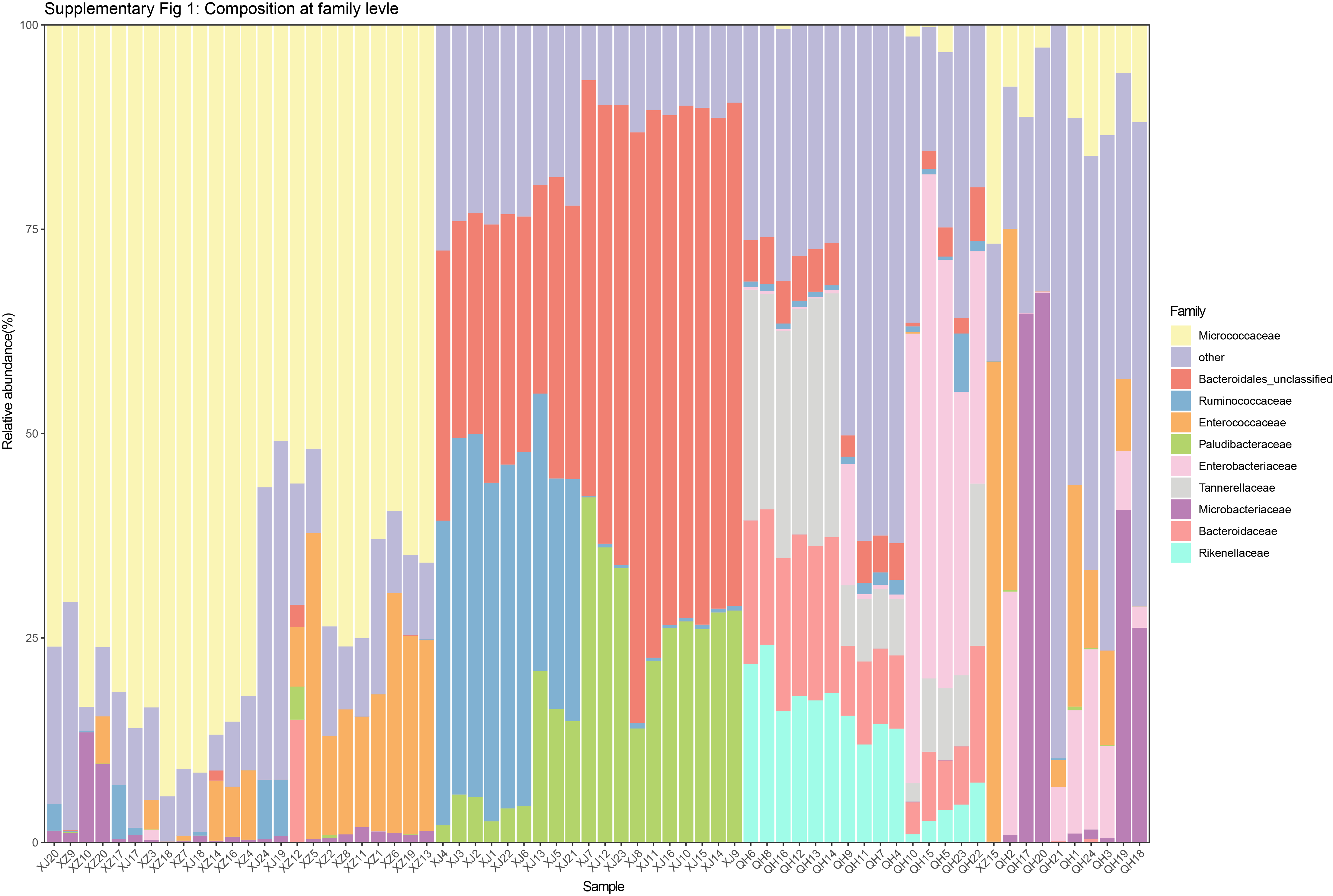

Supplement: Supplementary Figure 1 — Family-level composition of the Tibetan antelope gut microbiome. [file Image1.tif]

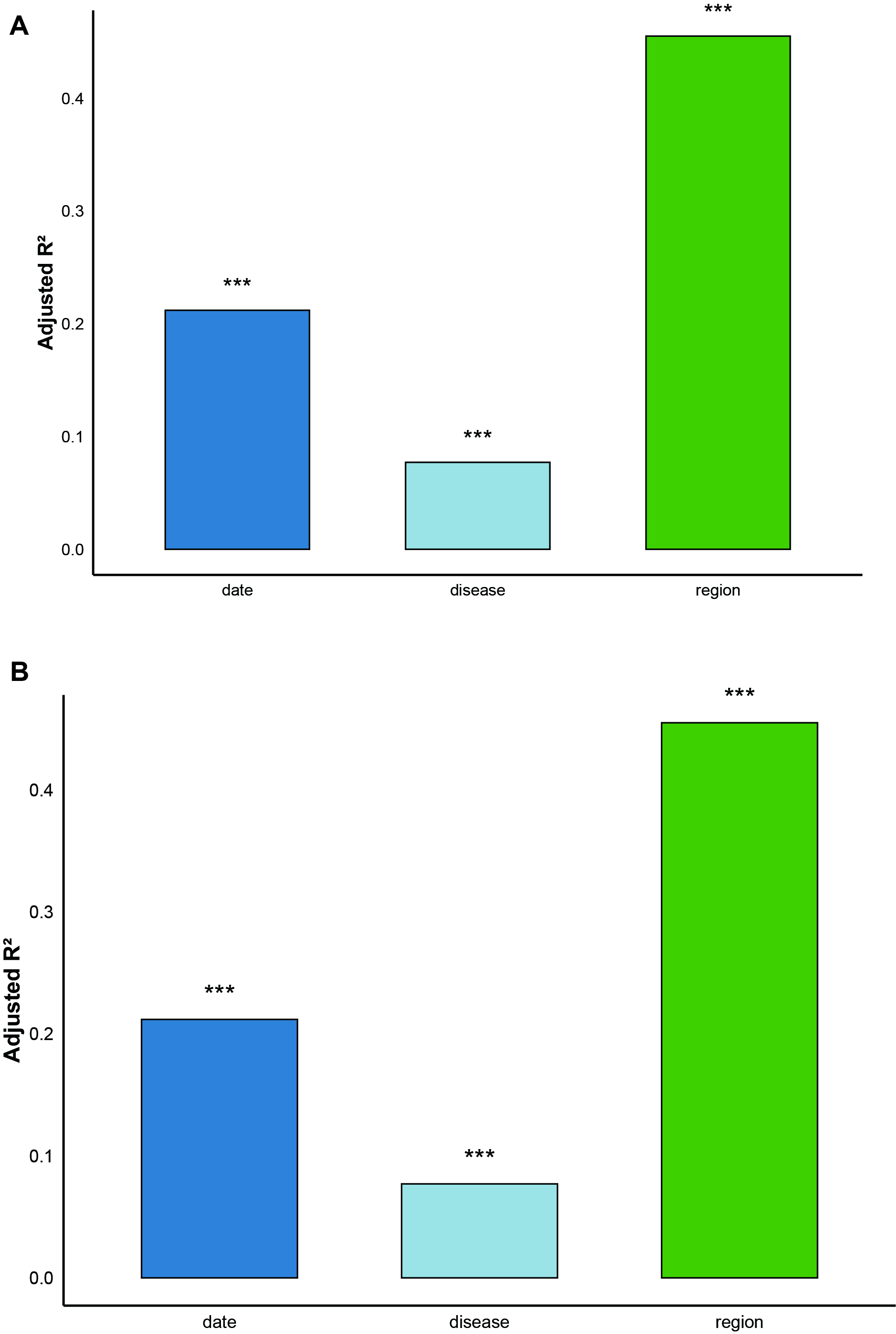

Supplement: Supplementary Figure 2 — Permutational multivariate analysis of variance (PERMANOVA) based on Bray – Curtis distance matrices. Two approaches were applied: (A) the marginal effects method (by=“margin”) to assess the independent contribution of each factor, and (B) the sequential method (by=“terms”) to evaluate the contributions of main effects and interactions. * P<0.05; ** P<0.01. [file Image2.tif]

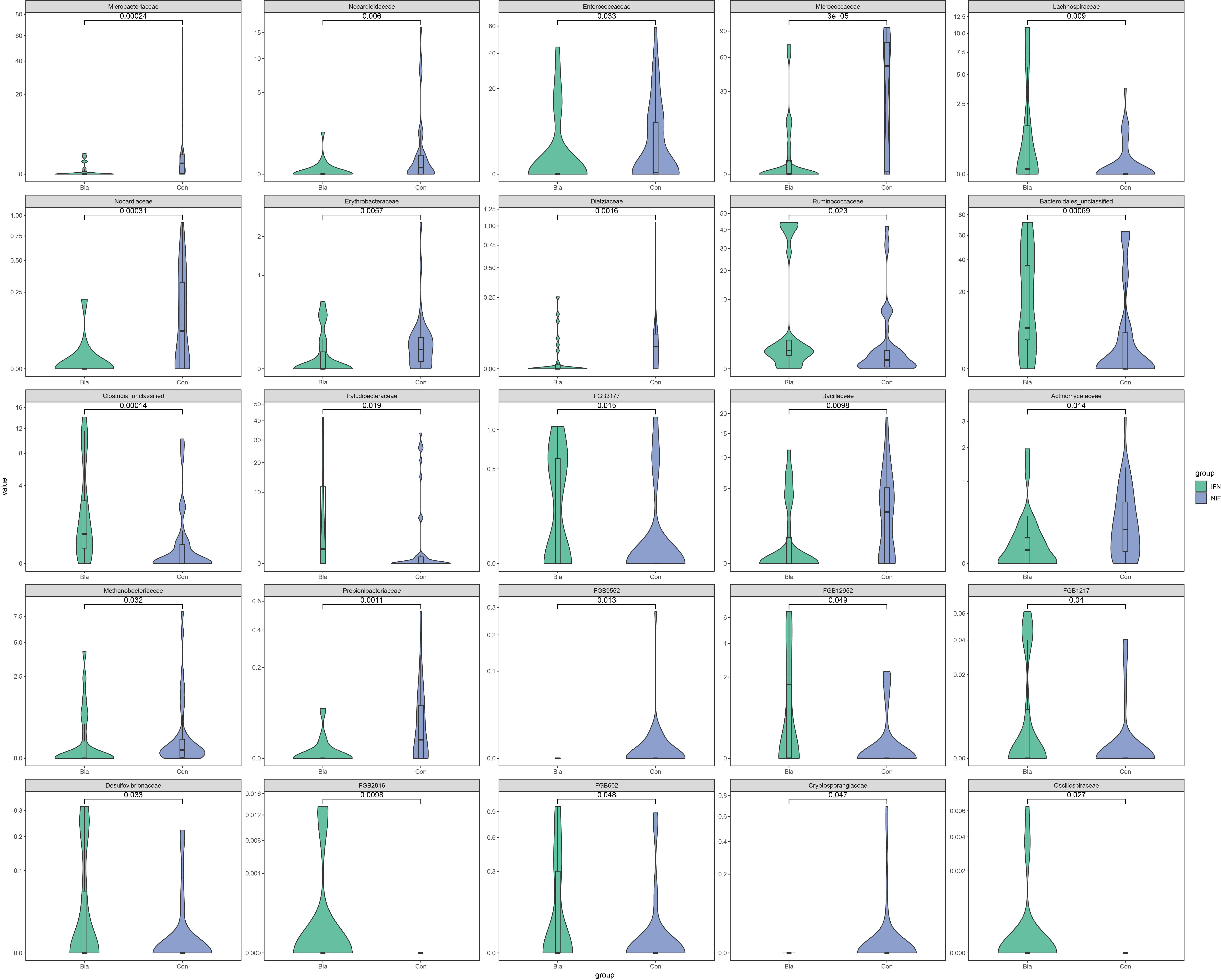

Supplement: Supplementary Figure 3 — Differential bacterial families (Wilcoxon p<0.05) between the infected and uninfected groups. [file Image3.tif]

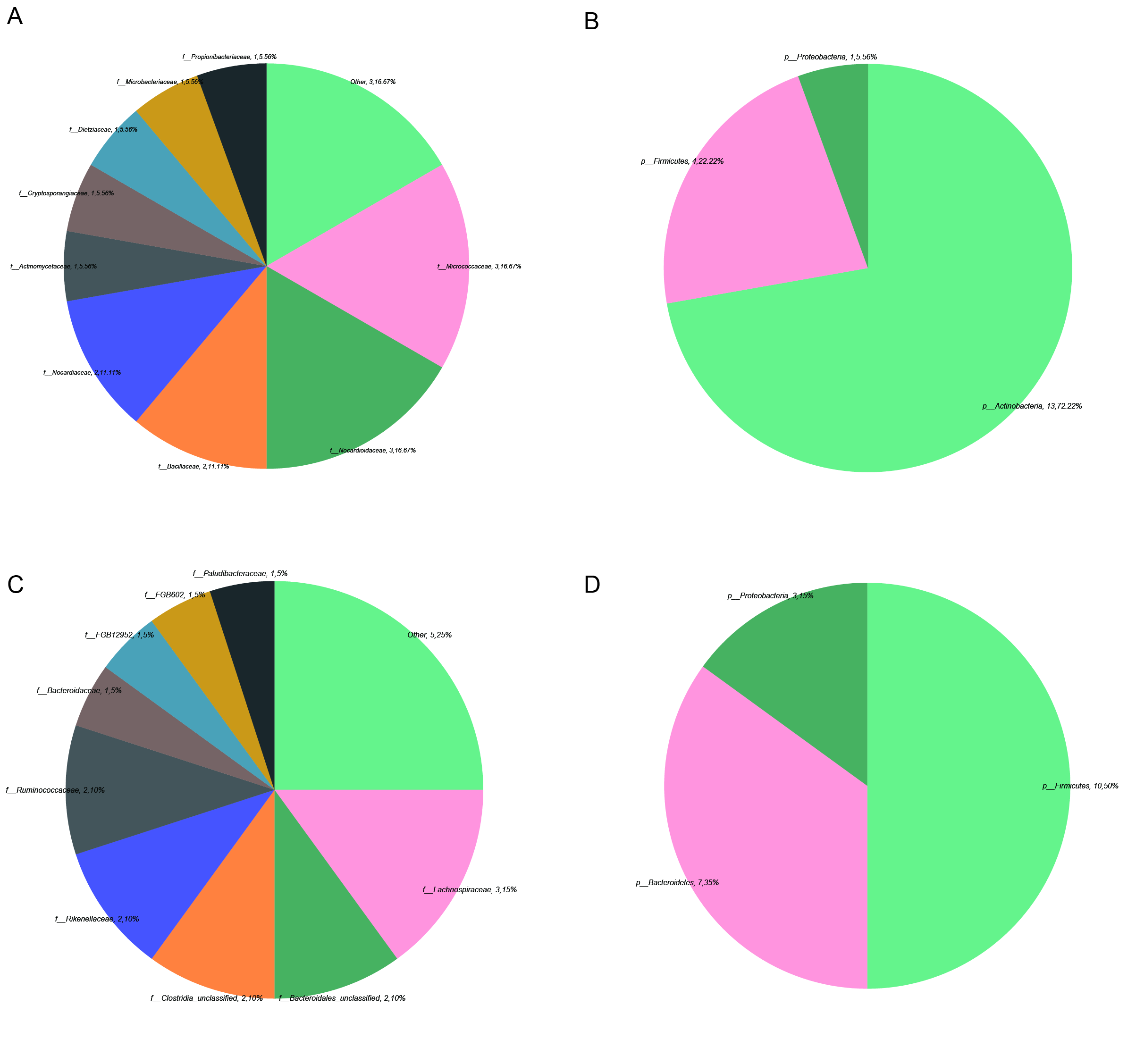

Supplement: Supplementary Figure 4 — Distribution proportions of differential bacteria at the family and phylum levels. (A-B) Uninfected group. (C-D) Infected group. [file Image4.tif]

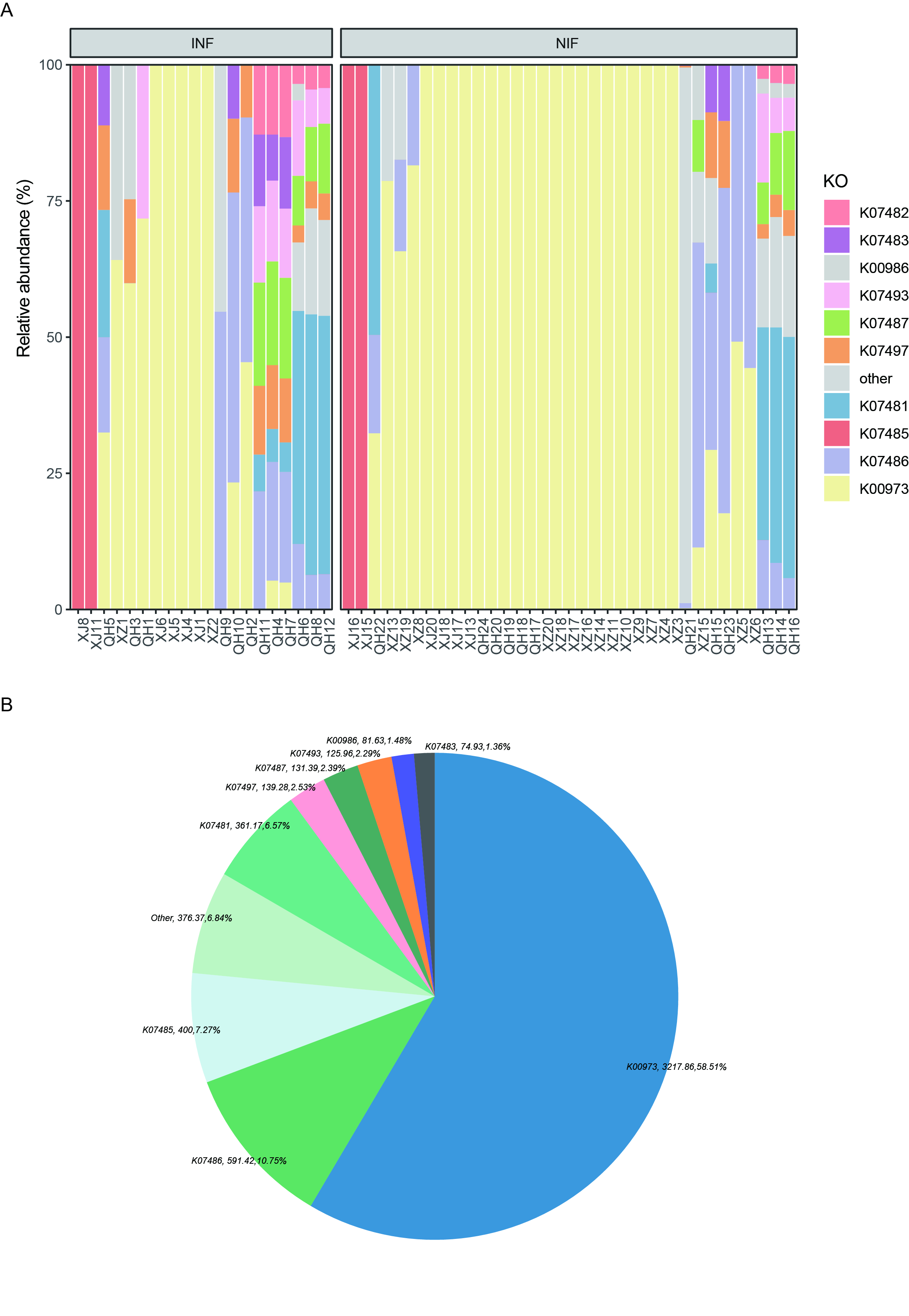

Supplement: Supplementary Figure 5 — KO composition. (A) Composition of relative abundances of KO pathways. (B) Overall composition proportions of KO pathways. [file Image5.tif]
